# Induction of Protective Immunity against *Toxoplasma gondii* in Mice by Nucleoside Triphosphate Hydrolase-II (NTPase-II) Self-amplifying RNA Vaccine Encapsulated in Lipid Nanoparticle (LNP)

**DOI:** 10.3389/fmicb.2017.00605

**Published:** 2017-04-05

**Authors:** Fangjun Luo, Lina Zheng, Yue Hu, Shuxian Liu, Yan Wang, Zhongkui Xiong, Xin Hu, Feng Tan

**Affiliations:** ^1^Department of Clinical Laboratory, Zhuji People’s HospitalZhuji, China; ^2^Institute of Stem Cell and Tissue Engineering, School and Hospital of Stomatology, Wenzhou Medical UniversityWenzhou, China; ^3^Department of Parasitology, School of Basic Medical Sciences, Wenzhou Medical UniversityWenzhou, China; ^4^Department of Radiation Oncology, Shaoxing Second HospitalShaoxing, China; ^5^Department of Basic Laboratory Medicine, School of Medical Laboratory Science and School of Life Science, Wenzhou Medical UniversityWenzhou, China

**Keywords:** *Toxoplasma gondii*, vaccine, nucleoside triphosphate hydrolase, self-amplifying RNA, lipid nanoparticle (LNP)

## Abstract

RNA-based vaccine represents an irresistible and safe immunization strategy with decreasing theoretical risks of genomic integration and malignant cell transformation. To our knowledge, however, there is no report about development of RNA vaccine against *Toxoplasma gondii* infection. We have previously demonstrated that the recombinant *T. gondii* nucleoside triphosphate hydrolase-II (NTPase-II) protein is able to provide protective Th1 cell-mediated immunity against *T. gondii.* Herein, we evaluated the immunogenic potential of a self-amplifying RNA vaccine-encoding *T. gondii* NTPase-II gene, RREP-NTPase-II, delivered by a synthetic lipid nanoparticle (LNP). Immunization of mice with naked RREP-NTPase-II induced a strong cellular and humoral immune response with high-IgG antibody titers and IFN-γ production. The immunized mice displayed significantly prolonged survival time and reduction in brain parasite load (46.4%) compared with control group. Furthermore, mice vaccinated with RREP-NTPase-II-encapsulated LNP displayed significantly enhanced protection against acute infection as well as chronic infection with PRU cyst, which shows 62.1% reduction in brain cyst burden in comparison to control group. These results suggest that the combination of self-amplifying RNA and LNP ion would be beneficial to the development of a safe and long-acting vaccine against toxoplasmosis.

## Introduction

*Toxoplasma gondii*, the pathogen of toxoplasmosis, is an obligate intracellular parasitic protozoan, which has a wide range of hosts including humans and warm-blooded animals (Cenci-Goga et al., 2011). Hosts could be infected through consumption of raw or undercooked meat of animals containing *Toxoplasma* cysts, or through ingestion of water or vegetables contaminated with *Toxoplasma* oocysts. Although *T. gondii* infection is usually asymptomatic in immunocompetent hosts, it is a serious threat to pregnant and immunocompromised individuals (Dubey, 2010).

Vaccines against *Toxoplasma* have been explored for a long time. However, ToxoVax, based on live attenuated S48 strain, is only one commercial vaccine for farm animals (Buxton and Innes, 1995). But it is unlikely to be applied to humans because of limitations of reduced efficacy as well as biosafety concerns (Zhang et al., 2013). To surmount this defect, current development trials of vaccines against *T. gondii* infection have been focused mainly on the subunit, recombinant, and nucleic acid vaccines (Jongert et al., 2009; Zhang et al., 2013). Among these different approaches, development of nucleic acid-based vaccine is a promising approach due to less expense, easiness to handle, as well as its ability to induce both humoral and cellular immune responses with low dose (Tang et al., 1992). To our knowledge, however, there is no report about development of RNA vaccine against *T. gondii* infection although plasmid-based DNA vaccines have been paid attention for several decades (Liu et al., 2012). The main obstacles to the development of RNA vaccine could be attributed to that RNA vaccine often elicits weak immune responses and requires multiple vaccinations because of the short intracellular half-life and easiness of degradation *in vivo* and during storage. Nonetheless, RNA-based vaccination still exhibits an irresistible advantage that RNA molecule exists solely in the cytoplasm, thereby extensively decreasing theoretical risks of genomic integration and malignant cell transformation, which give rise to safety concerns for DNA vaccines (Kofler et al., 2004). That is why RNA vaccination is not categorized as gene therapy by regulatory authorities. Thus far, the non-amplifying mRNA vaccines have been utilized in experimental animals for elicitation of humoral and cellular immune responses against tumor (Pascolo, 2008; Fotin-Mleczek et al., 2011), allergy (Weiss et al., 2012), and infectious disease (Lorenzi et al., 2010).

Recently, a self-amplifying RNA vector, pRREP, based on an alphavirus Semliki Forest virus (SFV) genome has been utilized to improve the weak immune responses induced by mRNA vaccines (Fleeton et al., 2001; Johansson et al., 2012). The skeleton of self-amplifying RNA mainly consists of the gene encoding the viral RNA replicase and the antigen of interest (AOI)-encoding mRNA, which replaces the viral structural protein gene. Upon transfection, the AOI would be plentifully expressed by the replicase complex amplification in the cytoplasm of the transfected cells (Karlsson and Liljestrom, 2004). In addition, this strategy avoids safety concerns and complicated operation because the RNA could be directly prepared by transcribing a linearized DNA plasmid using a T7 RNA polymerase *in vitro* (Johansson et al., 2012). Moreover, a synthetic lipid nanoparticle (LNP) delivery system has been utilized to deliver self-amplifying RNA in order to further enhance the vaccination efficiency *in vivo* (Geall et al., 2012; Hekele et al., 2013).

*Toxoplasma gondii* nucleoside triphosphate hydrolase (NTPase), accounting for 2–8% of the total protein of tachyzoites, has a potent apyrase activity and is released from dense granules into parasitophorous vacuole for successively degrading ATP to ADP and finally AMP (Asai et al., 1983; Nakaar et al., 1998). Two isoforms of NTPase have been verified in *T. gondii*. However, NTPase-I is expressed only in type I virulent strains, while NTPase-II generally exists in all strains (Asai et al., 1995). This difference indicates the possibility that NTPase-II might be more preferable as a potential vaccine candidate than NTPase-I, and this speculation is supported by the finding that the recombinant NTPase-II protein could elicit a strong specific Th1 immune response and partially protect the experimental mice from acute and chronic *Toxoplasma* infection (Tan et al., 2011).

In this study, we evaluated the potency of a self-amplifying RNA, RREP-NTPase-II, to induce specific immune response and protective efficiency anti-*Toxoplasma* challenge in BALB/c mice and tested further whether LNP delivery system effectively improves the immune response. We found that RREP-NTPase-II indeed elicited both humoral and cellular immune responses that could be enhanced by LNP encapsulation, indicating that the combination of self-amplifying RNA vaccine and LNP delivery system is a promising approach with an improved safety and immunogenicity profile.

## Materials and Methods

### Mice

Female BALB/c mice and ICR mice (6–8 weeks old) were purchased from Laboratory Animal Center, Wenzhou Medical University, Zhejiang, China. They were bred and handled in strict accordance with the Good Animal Practice requirements of the Animal ethics Procedures and Guidelines of China. This research was approved by the Laboratory Animal Ethics Committee of Wenzhou Medical University (Permit number: wydw2015-0073). Humane endpoints are considered to avoid the mice pain or suffering via euthanasia. Mice were monitored daily for signs of toxoplasmosis, such as fatigue, difficulty in feeding and severe ascites. Mice shown above signs would be sacrificed immediately with CO_2_ gas.

### Parasites

The cyst-forming type II strain PRU was maintained in female ICR mice. Forty-five days after infection, cysts were harvested from infected mouse brains for challenge of immunized mice. The virulent type I strain RH was routinely propagated in human foreskin fibroblast (HFF) as previously described (Li et al., 2016) and employed for acute challenge infection.

### Plasmid Construction and RNA Synthesis

**FIGURE 1:**
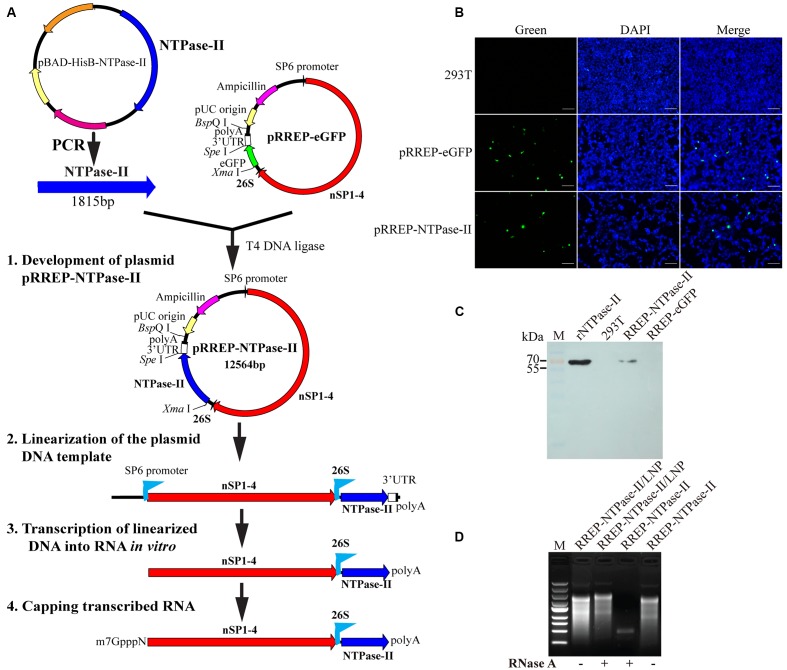
**Characterization of self-amplifying RNA. (A)** Flow diagram of development of a self-amplifying RNA vaccine, RREP-NTPase-II, based on an alphavirus Semliki Forest virus (SFV) RNA vector. polyA, polyadenylation signal; 26S, the subgenomic promoter of SFV; eGFP, enhanced green fluorescent protein gene; NTPase-II, the full CDS of *Toxoplasma gondii* NTPase-II gene without the signal peptide; nSP1–4, the non-structural protein genes of SFV. **(B)** Immunofluorescence assay (IFA). The self-amplifying RNA was transiently transfected into 293T cells and the expression of NTPase-II was identified by IFA using specific-NTPase-II monoclonal antibody. Scale bars, 100 μm. **(C)** Western blot analysis. Cell lysates were separated by SDS–PAGE and NTPase-II was visualized using anti-NTPase-II monoclonal antibody. Recombinant NTPase-II (rNTPase-II) was loaded as positive control, cell lysates from either non-transfected cells (293T) or transfected with RREP-eGFP (RREP-eGFP) as negative control. **(D)** RNase protection evaluation. Before and after exposure to RNase A, RNA from either naked self-amplifying RNA or self-amplifying RNA encapsulated with lipid nanoparticle (LNP) was extracted and analyzed by RNA agarose gel electrophoresis.

The DNA sequence of NTPase-II gene was amplified from the construct pBAD-HisB-NTPase-II (Tan et al., 2011) using a set of specific primers 5′-TTCCCGGGATGACAGACTCATCGTCACTCC-3′ and 5′-CCACTAGTTCACAGATTGTGAGAATATCCCGCC-3′ and was inserted into *Xma* I and *Spe* I site of pRREP-eGFP (Karlsson and Liljestrom, 2004) to swap the coding sequence of eGFP. The resulting plasmid, pRREP-NTPase-II, was purified with E.Z.N.A.^®^ Plasmid Mini Kit (OMEGA Bio-tek, USA) and stored at -20°C. Prior to immunization, the plasmids were linearized and RNAs *in vitro* transcription were made by using MEGAScript^TM^ Kit (Ambion, USA) as described previously (Geall et al., 2012). Finally, the purified transcribed RNAs were capped with Vaccinia Capping System (NEB, USA) before encapsulation (**Figure 1A**).

### Detection of Expression of NTPase-II *In vitro*

The self-amplifying RNA was transfected into 293T cells at once post-transcription according to the manufacturer’s instructions of Lipofectamine^®^ 2000 Reagent (Invitrogen, USA). Twenty-four hours after transfection, expression of NTPase-II was confirmed by indirect immunofluorescence assay (IFA) with the mouse monoclonal antibody anti-NTPase-II (1:2000 dilution) that has been raised in our laboratory (Tan et al., 2010) as the primary antibody. Finally, cells were stained with 0.5 μg/mL DAPI for 10 min and the fluorescent images were obtained with a fluorescence microscope (Nikon, Japan). Additionally, the expression of NTPase-II was further analyzed by Western blot using anti-NTPase-II monoclonal antibody. Cell lysates from either non-transfected or RREP-eGFP-transfected cells were used as a negative control, recombinant NTPase-II was used as a positive control.

### Lipid Nanoparticle (LNP) Encapsulation of RNA

The preparation of LNP and encapsulation of RNA were identical with the previous description (Geall et al., 2012; Ma et al., 2014). Briefly, the LNP, consisting of 1,2-distearoyl-*sn*-glycero-3-phosphocholine (DSPC, Sigma–Aldrich, USA), cholesterol (Sigma–Aldrich), 1,2-dimyristoyl-*sn*-glycero-3-phosphoethanolamine-*N*-[methoxy (polyethylene glycol)-2000] (ammonium salt) (PEG DMG 2000) (Avanti Polar Lipids, USA), and 1,2-dilinoleyloxy-3-dimethylaminopropane (DLinDMA) synthesized as reported previously (Heyes et al., 2005) was produced by ethanol dilution process at 10:48:2:40 molar ratio (Geall et al., 2012). Then, the self-amplifying RNA vaccines dissolved in 100 mM citrate buffer were encapsulated in LNP by spontaneous vesicle formation process as previously described (Ma et al., 2014). The resulting RNA/LNP was dialyzed against PBS overnight at 4°C and finally sterile-filtered through a 0.22 μm filter. Prior to vaccination, samples were diluted to the indicated RNA concentration with PBS.

### Evaluation of Encapsulation Efficiency and RNase Protection

Ribonucleic acid/lipid nanoparticle formulations were characterized for encapsulation efficiency and the ability to protect the encapsulated RNA from RNase as previously described (Geall et al., 2012). To determine the encapsulation efficiency, the percentages of encapsulated RNA were determined by detecting the RNA concentrations of both outside and inside the LNP using Quant-iT^TM^ RiboGreen^®^ RNA Assay Kit (Invitrogen, USA) according to the manufacturer’s instructions. Briefly, RNA/LNP samples were diluted in TE buffer prior to addition of the dye, and the RNA concentrations outside the LNP were determined by detecting the fluorescence with a microplate reader (BioTek, USA). Afterward, the total RNA concentrations, including the RNA outside and inside LNPs, were obtained after the lipid membranes were breached using Triton X-100. To evaluate RNase protection of the encapsulated RNA, the RNA/LNP samples were treated with RNase A (Invitrogen, USA) for 30 min. After RNase inactivation with proteinase K (Invitrogen, USA), RNA was extracted and denaturing agarose gel electrophoresis was performed.

### Immunization

One hundred 6- to 8-week-old female BALB/c mice were divided into 5 groups with 20 mice per group. They were injected intramuscularly into bilateral quadriceps with 10 μg of vaccines diluted in PBS on day 0 and 21. Mice that received PBS were used as non-immunized control. For the determination of specific antibodies, serum samples from all groups were obtained 14 days after the last immunization. For the analysis of cellular immune responses, 5 spleen samples from each group were aseptically collected on day 35.

### Evaluation of Humoral Response

Specific anti-NTPase-II IgG antibody levels were measured by ELISA according to our previous description (Tan et al., 2011) with minor modifications. In brief, ELISA plates (Corning, USA) were coated overnight at 4°C with 5 μg/mL purified recombinant NTPase-II protein. After washing three times with PBS plus 0.05% Tween 20 (PBST), the coated plates were blocked with 5% skim milk in PBST for at least 1 h at 37°C. Afterward, 100 μL of twofold serial dilution of serum samples from 1:100 in PBST solution were added and incubated for 1 h at 37°C. After washing, specific anti-NTPase-II total IgG or IgG isotype were detected by incubating plates with 100 μl of HRP-conjugated rabbit anti-mouse IgG, IgG1, or IgG2a (Invitrogen, USA) for 1 h at 37°C. The plate was washed extensively, and incubated with 100 μl of 3,3,5,5-tetramethylbenzidine (TMB). The reaction was stopped by adding 50 μl of 2.5 N H_2_SO_4_ after 10 min, and the optical density at 450 nm was detected with a microplate reader. Endpoint titers were calculated or at the dilution when the absorbance fell below 0.2.

### Evaluation of Cellular Response

Two weeks after the last booster immunization, five mice per group were sacrificed to obtain single splenocyte suspensions as described previously (Tan et al., 2011). After erythrocytes were lysed, the splenocytes were resuspended and plated in triplicate in 96-well plates (3 × 10^5^ cells/well) in presence of 10 μg/ml recombinant NTPase-II proteins or in plain complete medium as negative control or 5 μg/ml ConA as positive control. Plates were incubated for 72 or 96 h at 37°C and 5% CO_2_.

For lymphocyte proliferation assay, the stimulated splenocytes were cultured for 72 h, and the spleen lymphocyte proliferative activity was detected with CCK-8 reagent (Dojindo Laboratories; Kumamoto, Japan) in accordance to the manufacturer’s instruction. The absorbance at 450 nm was read, and then, the stimulation index (SI) was calculated as the ratio of the average OD_450_ value from recombinant NTPase-II-stimulated cultures/the average OD_450_ value from cells with medium cultures.

For IFN-γ analysis, cell-free supernatants from the stimulated splenocytes were collected and the IFN-γ activity was analyzed at 96 h after stimulation. According to the manufacturer’s instructions of Mouse IFN-γ ELISA Ready-SET-Go!^®^ (eBioscience, USA), their IFN-γ concentrations were determined by reference to a standard curve constructed with known amounts of mouse IFN-γ and the sensitivity limit was 15 pg/ml.

### Challenge Infection

Two weeks after the last booster immunization, five mice per group were infected intraperitoneally with 10^3^ tachyzoites of RH strain and observed for an additional 30 days. The survival time and the percentages of mice survived were recorded daily.

In addition, 10 mice of each group were orally challenged with 20 tissue cysts of PRU strain on day 35. Mortality was observed daily for 45 days, and the surviving mice were sacrificed for quantification of cysts in brain as described previously (Tan et al., 2011).

### Statistical Analysis

Statistical analysis was carried out with one-way ANOVA and multiple comparison procedures using SPSS 13.0 software for each assay, and Kaplan–Meier test for survival analysis. Data were represented as the mean ± SEM. Differences were considered statistically significant when *p* < 0.05.

## Results

### Detection of Expression of NTPase-II by IFA

Plasmid pRREP-NTPase-II as templates for transcription of RNA was constructed successfully by verification with sequencing (data not shown). For the assessment of protein expression *in vitro*, transfection of self-amplifying RNA into 293T cells was performed at once post-transcription and capping. At 24 h post-transfection, expression of the NTPase-II protein was verified by IFA with specific monoclonal antibody. The fluorescence was observed in the cells transfected with self-amplifying RNA, whereas no fluorescent signal was observed in the negative control (**Figure 1B**), demonstrating that NTPase-II protein could be expressed in mammalian cells. The identity of the expressed NTPase-II protein was further confirmed by Western blot. A single protein band was detected at the size of about 70 kDa corresponding to the predicted size of NTPase-II protein (**Figure 1C**).

### RNA/LNP Vaccine Characterization

After RNA was encapsulated in LNP, characteristics of RREP-NTPase-II/LNP formulations were analyzed. For the assessment of encapsulation efficiency, the concentrations of both free RNA and total RNA in solution post-encapsulation were measured. The result showed that ∼80% of the RNA was encapsulated. In addition, the integrity of encapsulated RNA was validated by denaturing agarose gel electrophoresis after treatment with RNase A (**Figure 1D**). The result showed that the encapsulated RNA was maintained, while the naked RNA was degraded by RNase A, demonstrating that the LNP has the ability to protect the encapsulated RNA from degradation.

### Evaluation of the Humoral Immune Response

**FIGURE 2:**
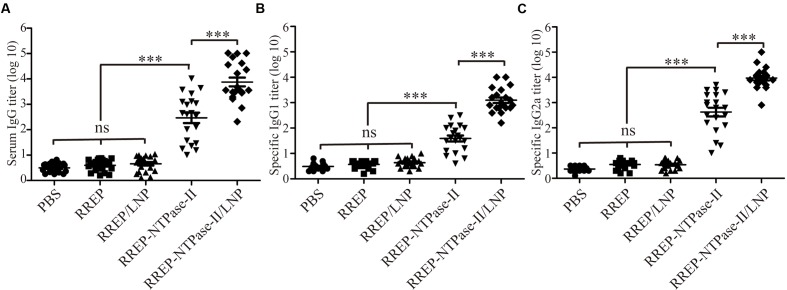
**Determination of specific humoral response in the immunized mice**. Two weeks after immunization, sera were obtained and NTPase-II-specific total IgG **(A)**, IgG1 **(B)**, and IgG2a **(C)** titers were determined by ELISA. Data are plotted as end point titers and shown for each individual mouse. Results are expressed as the mean of log_10_ titers ± SEM and are representative of at least two independent experiments. ^∗∗∗^*p* < 0.001; ns, not significant.

In order to evaluate if the immunization with self-amplifying RNA vaccine encoding NTPase-II antigen induced specific humoral immune response, the antibody titers in serum samples from immunized mice were detected by indirect ELISA 2 weeks after last vaccination. Results were presented as the mean of log_10_ titers ± SEM. As a result, a significantly higher NTPase-II-specific IgG titers were observed in groups immunized with RREP-NTPase-II (2.466 ± 0.206, *p* < 0.001) in contrast to three control groups, PBS (0.495 ± 0.040), RREP (0.60 ± 0.07), and RREP/LNP (0.656 ± 0.069; **Figure 2A**). In an additional experiment, the distribution of both IgG1 and IgG2a was also analyzed (**Figures 2B,C**). The levels of both IgG subtypes were significantly increased in mice immunized with RREP-NTPase-II (*p* < 0.001). Noteworthy, RREP-NTPase-II vaccine elicited slightly higher specific IgG2a (2.625 ± 0.174) levels when compared to IgG1 (1.59 ± 0.019), consistent with a Th1 response.

Furthermore, to investigate if LNP improved the humoral immune responses, mice were immunized with 10 μg of encapsulated RNA (RREP-NTPase-II/LNP). The encapsulated RNA elicited significantly higher total IgG (3.879 ± 0.173, *p* < 0.001) as well as both IgG subtypes titers, IgG1 (3.095 ± 0.106) and IgG2a (3.970 ± 0.094), than naked RNA (RREP-NTPase-II). Similarly, a Th1 response was elicited in immunized mice. These results indicate that the presence of LNP enhances the specific humoral immune response.

### Evaluation of the Cellular Immune Responses

**FIGURE 3:**
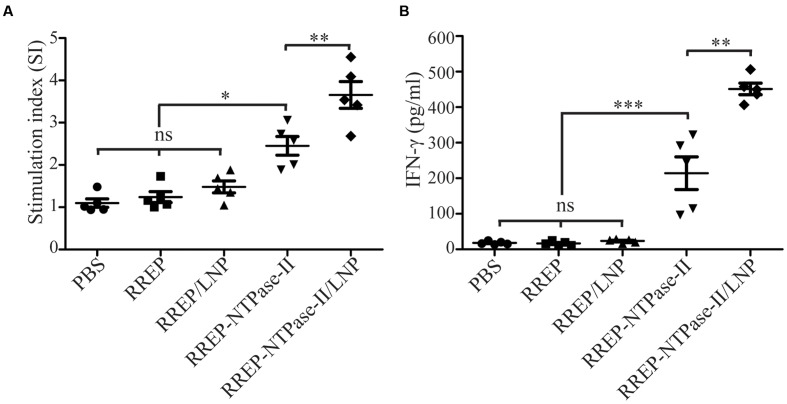
**Cellular responses in splenocytes isolated from the immunized mice**. Two weeks after immunization, splenocytes were harvested from five mice per group. After stimulation with recombinant NTPase-II (10 μg/mL), cellular immune responses were analyzed. **(A)** Lymphocyte proliferative assay. Seventy-two hours after stimulation, the lymphocyte proliferative response was detected and expressed as stimulation index (SI). **(B)** IFN-γ production. Supernatants of stimulated splenocytes were assessed by ELISA for the production of IFN-γ at 96 h after stimulation. Data represent the mean ± SEM and are representative of at least two independent experiments. ^∗^*p* < 0.05, ^∗∗^*p* < 0.01, and ^∗∗∗^*p* < 0.001; ns, not significant.

Two weeks after last vaccination, splenocytes were harvested from immunized mice and an *in vitro* lymphocyte proliferation assay was performed. Both RREP-NTPase-II (2.454 ± 0.221, *p* < 0.001) and RREP-NTPase-II/LNP (3.656 ± 0.317, *p* < 0.001) elicited a significantly higher SI values to the NTPase-II antigen stimulation. On the contrary, administration with PBS (1.098 ± 0.100), RREP (1.240 ± 0.129), or RREP/LNP (1.482 ± 0.143) induced little lymphocyte proliferation to NTPase-II stimulation (**Figure 3A**).

To further determine if immunization with self-amplifying RNA vaccine induced a cellular immune response, the concentration of IFN-γ in supernatants of splenocytes stimulated with NTPase-II antigen was measured. Upon stimulation, RREP-NTPase-II (214.10 ± 46.01) produced significantly higher levels of IFN-γ (*p* < 0.001). For RREP-NTPase-II/LNP (451.34 ± 16.26), the positive effect was even more pronounced and increased twofold than those from RREP-NTPase-II (*p* < 0.01). In contrast, neither the PBS control group (18.05 ± 1.98) nor the groups that immunized with RREP (16.59 ± 2.75) or RREP/LNP (23.64 ± 2.29) developed any detectable amount of IFN-γ (**Figure 3B**). These results suggest that RNA/LNP was able to elicit the antigen-specific IFN-γ-mediated responses.

### Assessment of Protective Efficacy Against *T. gondii* Infection

**FIGURE 4:**
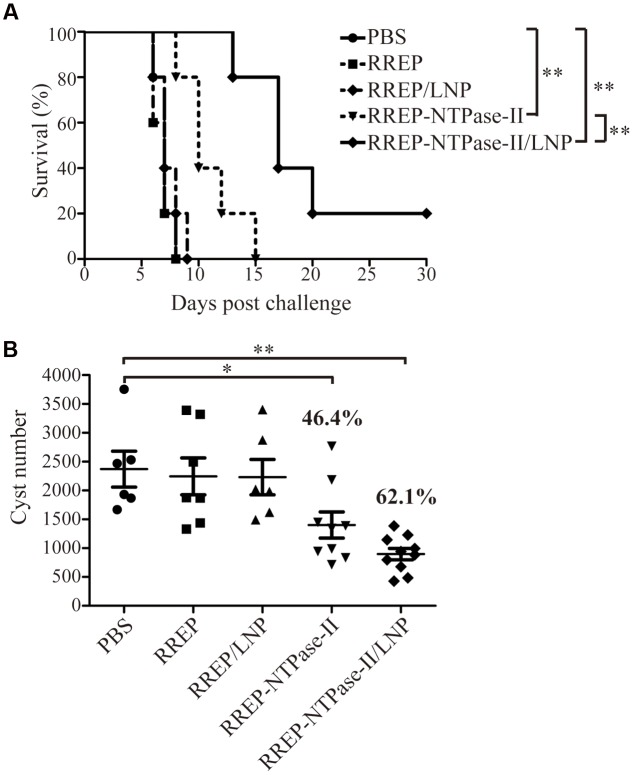
**Protection of BALB/c mice against *T. gondii* infection. (A)** Survival rate of vaccinated mice after RH strain tachyzoites challenge. Two weeks later post-immunization, five mice per group were intraperitoneally infected with 10^3^ live RH strain tachyzoites and observed daily for mortality. The final survival rates were calculated, ^∗∗^*p* < 0.01. **(B)** The cyst number in brain after PRU strain cyst challenge. Another 10 mice per group were challenged by gavage with 20 PRU strain cysts; 45 days after challenge, brain cysts were counted in surviving mice. Data represent the group mean ± SEM. The results presented are representative of two independent experiments. ^∗^*p* < 0.05 and ^∗∗^*p* < 0.01.

To evaluate if self-amplifying RNA vaccine could confer effective protection against *T. gondii* acute infection, five mice per group were challenged with 10^3^ RH strain tachyzoites 2 weeks after boosting (**Figure 4A**). All mice immunized with PBS, RREP, or RREP/LNP died within 9 days after challenge. However, a significantly prolonged survival time was observed in mice immunized with both RREP-NTPase-II and RREP-NTPase-II/LNP (*p* < 0.01). Especially in the RREP-NTPase-II/LNP group, one mouse still survived after 30 days of challenge, which showed the highest survival rate and was significantly higher than that of the RREP-NTPase-II group (*p* < 0.01).

Furthermore, the resistance to *T. gondii* chronic infection was also evaluated. The immunized mice were challenged with 20 PRU strain cysts 2 weeks after last immunization (**Figure 4B**). Forty-five days after the challenge, direct cyst counting was performed by optical microscopy in the brains of survived mice. Similar to the results of acute challenge, the number of brain cysts showed a significant reduction in both RREP-NTPase-II by 46.4% (1402.00 ± 225.50, *p* < 0.05) and RREP-NTPase-II/LNP by 62.1% (897.30 ± 98.22, *p* < 0.01) compared with PBS (2370.17 ± 310.36), RREP (2245.86 ± 319.89), or RREP/LNP (2231.67 ± 306.02). Therefore, immunization with self-amplifying NTPase-II RNA encapsulated with LNP enhanced a substantial resistance to the acute and chronic challenges of *T. gondii.*

## Discussion

This study applies a RNA/LNP vaccination strategy, explored for inducing protective immunity against viral infection, to induce effective immune responses against *T. gondii* challenge. Significant information obtained demonstrates that a potent immune response could be induced by self-amplifying RNA, and this response can be further enhanced by LNP delivery system.

Currently, there is a popular view that the nuclear membrane of transfected cells is attributed to the major barrier for restriction of delivering DNA vaccine into nuclear and consequently reduction of the expression level of AOI (Dupuis et al., 2000). Particularly, it seems to be ineffective in non-dividing cells, such as mature myocytes. Therefore, while many DNA vaccines are able to induce strong immune responses in small animals, the immunity is generally weaker in larger animals, with the amount of DNA vaccine required for efficient immunity being 1,000-fold higher in larger species than in small animals (Kutzler and Weiner, 2008). In addition, although this limitation can be surmounted by immunizing more DNA and exploiting electroporation to promote the DNA transport from cytoplasm into nuclear (Dupuis et al., 2000), anti-vector immune response and hazard of vector chromosomal integration must be considered (Smerdou and Liljestrom, 1999; Kofler et al., 2004). On the contrary, self-amplifying RNA vaccine employed in the present study circumvents these limitations due to its characteristics of strict cytoplasmic replication and expression, which improves the transfection efficient in different cell types, diminishes the need for codon modification, and excludes the risks of splicing and degradation (Johansson et al., 2012). To date, the vaccination of both naked or liposome-encapsulated non-amplifying mRNA (Weide et al., 2008, 2009; Rittig et al., 2011) and self-amplifying RNA packaged in viral replicon particles (VRPs; Bernstein et al., 2009) has been validated in human clinical trials for safety as well as increased cellular or humoral immunity. Herein, we have successfully constructed the self-amplifying RNA plasmid vaccine, pRREP-NTPase-II, and the expression of NTPase-II was verified by IFA and Western blot. Although we did not directly make a comparison between the self-amplifying RNA vaccine and DNA vaccine, we reasonably believe the self-amplifying RNA vaccine has several advantages than DNA vaccine because the lower dose of self-amplifying RNA vaccine (10 μg/time) and the less time of immunization (two times) can still elicit significant immune response in comparison with the conventional DNA vaccine, usually requires more than 100 μg plasmid per time and at least three times (Angus et al., 2000; Vercammen et al., 2000; Yuan et al., 2011).

In addition, in our study, the production and amplification of DNA templates for *in vitro* transcription were completed using standard molecular cloning techniques. The self-amplifying RNA was then transcribed *in vitro* using a commercial kit, which can produce up to milligram quantities of RNA. Compared with other mRNA vaccine, the production of the self-amplifying RNA not only eliminates the potential risks that produce infectious virus via recombination, but circumvents the immunity against viral vectors because the viral replicon is intrinsically able to induce apoptosis of the transfected cell, resulting in transient and self-eliminating expression of the self-amplifying RNA vaccine (Johanning et al., 1995; Berglund et al., 1998; Fleeton et al., 2001). Importantly, the self-amplifying RNA is able to induce both humoral and cellular immune responses as determined in the present study.

Currently, many kinds of adjuvants have been applied and reported to enhance immune responses and protection against *T. gondii* in a good deal of studies. However, to our knowledge, the LNP delivery system is not so far utilized as an adjuvant in the development of vaccines against *T. gondii*, even though it has been developed to encapsulate self-amplifying mRNA for eliciting specific immune responses against antigens from HIV (Geall et al., 2012) or influenza virus (Hekele et al., 2013). In these studies, they have shown that the LNP encapsulation dramatically improves the potency of the self-amplifying RNA because the LNP delivery system might enhance transfection efficiency, prevent the RNA degradation from enzymes at the injection site, as well as boost antigen presenting cells to sequester RNA/LNP particles, thereby promoting antigen production and stimulating the innate immunity within these immune cells. In the present study, we have successfully encapsulated the self-amplifying RNA with LNP by an ethanol dilution process. Agarose gel electrophoresis determined that LNPs provide an effective protection of self-amplifying RNA from RNase A degradation.

As an obligate intracellular parasitic protozoan, Th1 cell-mediated immunity is considered to play a critical role in protective immunity to *T. gondii* (Jongert et al., 2009, 2010). IFN-γ, one of Th1-type cytokines, has been proven to be a pivotal mediator of resistance to *Toxoplasma in vivo* (Suzuki et al., 1988). Our previous studies (Tan et al., 2011) have demonstrated that the recombinant NTPase-II protein is able to provide protective Th1 cell-mediated immunity against *Toxoplasma* by induction of lymphocyte proliferation, high IFN-γ production, and activation of CD8+ T cells, the major T cell subset associated with acquired immune protection against *T. gondii* (Suzuki et al., 1989; Tan et al., 2011). Therefore, we paid more attention to assess the extent of cellular immune response using spleen lymphocytes from immunized mice. These results validated the significantly increased lymphocyte proliferation and IFN-γ level to *Toxoplasma* NTPase-II protein in mice immunized with naked self-amplifying RNA. More importantly, the enhanced lymphocyte proliferation response and higher IFN-γ production were promoted by LNP encapsulation. These findings indicate that immunization with naked self-amplifying RNA or LNP-encapsulated RNA indeed induces the IFN-γ-related Th1-type immunity, requiring for prevention of *Toxoplasma* infection, although more experiments should be designed to further evaluate the Th1-type immunity. Nevertheless, it is worth noticing that the immunity induced by RNA/LNP elicits a considerably higher protection rate than the naked self-amplifying RNA in experimental mice against *Toxoplasma* RH strain, significantly prolonging their surviving time up to 30 days after challenge. As a result, the higher survival rate and prolonged survival days further support that LNP encapsulation is better in inducing strong NTPase-II-specific IFN-γ production to sustain a protective immune response against *T. gondii*.

In addition, one advantage of self-amplifying RNA is the ability to elicit a significantly cellular memory immune response (Johansson et al., 2012). Although the memory T-cell immune responses were not investigated in the present study, the better control of brain cyst burden in both self-amplifying RNA-vaccinated mice groups during the chronic stage of toxoplasmosis, which is dependent on long-acting memory T cells, encourages us to speculate that self-amplifying RNA has the capability of eliciting an increase in effector memory T cells and the effect can be enhanced by LNP encapsulation. Also, the result consists with our previous finding that a significant decrease in brain cyst burden can be observed in mice vaccinated with NTPase-II protein, suggesting that *Toxoplasma* NTPase-II could be a potential vaccine candidate (Tan et al., 2011).

Although now it is not entirely clear to what extent the function of IgG antibodies is in anti-*Toxoplasma* infection, a widespread theory is that the specific IgG antibodies play a partial role in the resistance to *T. gondii* during secondary infection (Kang et al., 2000; Johnson and Sayles, 2002). In this study, therefore, the specific IgG antibodies were also detected in order to evaluate humoral immunity elicited by RNA vaccine. Expectedly, an enhanced anti-NTPase-II IgG response detected in sera from mice immunized with either naked self-amplifying RNA or RNA/LNP was validated. Furthermore, the serum IgG isotype profile was predominated by IgG2a, on behalf of a stronger Th1 cell-mediated immune responses, indicating that systemic humoral immunity elicited with RNA vaccine may involve in the prevention of *T. gondii* infection. More importantly, the IgG titers after vaccination with the RNA/LNP were superior to those induced by vaccination of naked RNA, which is consistent with previous findings that LNP encapsulation promotes a stronger Th1 cell response (Geall et al., 2012).

In summary, we have successfully encapsulated self-amplifying *T. gondii* NTPase-II RNA with LNP to produce RNA/LNP that can prevent the RNA degradation from RNase A. The naked self-amplifying RNA is capable to induce the protective NTPase-II-specific humoral and cellular immune responses to protect mice from *T. gondii* challenge. Additionally, RNA/LNP administered in the mouse enhances further the protective immunity. Noteworthily, although the partial protection was provided by this vaccine candidate, other effective immunization strategies and multi-antigens constructs should be included to produce better protective immunity in the future. In any case, the capability of the combination of self-amplifying RNA and LNP encapsulation to successfully elicit and extend protective immune response would be beneficial to the development of a safe and long-acting vaccine against *T. gondii* for future use in animals and humans.

## Author Contributions

FT conceived and designed the study. LZ and YH carried out plasmid construction and RNA synthesis. ZX prepared the lipid nanoparticle and encapsulated self-amplifying RNA. FL, SL, and YW performed immunization of mice and evaluation of protective immunity. FL and LZ analyzed the data and drafted the manuscript. XH and FT critically revised the manuscript. All authors read and approved the final manuscript.

## Conflict of Interest Statement

The authors declare that the research was conducted in the absence of any commercial or financial relationships that could be construed as a potential conflict of interest.
